# Perspectives on interstitial photodynamic therapy for malignant tumors

**DOI:** 10.1117/1.JBO.26.7.070604

**Published:** 2021-07-23

**Authors:** Katarzyna Komolibus, Carl Fisher, Johannes Swartling, Sune Svanberg, Katarina Svanberg, Stefan Andersson-Engels

**Affiliations:** a Tyndall National Institute, Biophotonics@Tyndall, IPIC, Cork, Ireland; b SpectraCure AB, Lund, Sweden; c Lund University, Department of Physics, Lund, Sweden; d South China Normal University, South China Academy of Advanced Optoelectronics, Guangzhou, China; e Lund University Hospital, Department of Clinical Sciences, Lund, Sweden; f University College Cork, Department of Physics, Cork, Ireland

**Keywords:** interstitial photodynamic therapy, solid tumors, dosimetry, photosensitizers

## Abstract

**Significance:** Despite remarkable advances in the core modalities used in combating cancer, malignant diseases remain the second largest cause of death globally. Interstitial photodynamic therapy (IPDT) has emerged as an alternative approach for the treatment of solid tumors.

**Aim:** The aim of our study is to outline the advancements in IPDT in recent years and provide our vision for the inclusion of IPDT in standard-of-care (SoC) treatment guidelines of specific malignant diseases.

**Approach:** First, the SoC treatment for solid tumors is described, and the attractive properties of IPDT are presented. Second, the application of IPDT for selected types of tumors is discussed. Finally, future opportunities are considered.

**Results:** Strong research efforts in academic, clinical, and industrial settings have led to significant improvements in the current implementation of IPDT, and these studies have demonstrated the unique advantages of this modality for the treatment of solid tumors. It is envisioned that further randomized prospective clinical trials and treatment optimization will enable a wide acceptance of IPDT in the clinical community and inclusion in SoC guidelines for well-defined clinical indications.

**Conclusions:** The minimally invasive nature of this treatment modality combined with the relatively mild side effects makes IPDT a compelling alternative option for treatment in a number of clinical applications. The adaptability of this technique provides many opportunities to both optimize and personalize the treatment.

## 1 Introduction

Malignant diseases are the second most common cause of death worldwide.^1^ The incidence rate is expected to increase by 47% by 2040, reflecting both growth and aging of the population as well as changes in other risk factors associated with socioeconomic development.^2^ In the combat of malignant diseases, there are presently three dominating and well-developed treatment modalities: surgery, chemotherapy, and radiation therapy, whereas other modalities such as immunotherapy^3^^,^^4^ are receiving increased attention.

The main local treatment modality for solid tumors is surgical resection. If possible, the entire tumor with a safety margin is removed during such resection. It is, however, in many cases, impossible to ensure the removal of all malignant cells. In these cases, the surgical resection is supplemented with either systemic or other localized treatments to decrease the probability of tumor recurrence. The most prominent systemic treatment modality for malignant diseases is chemotherapy.^5^ Chemotherapy relies on the administration of cytotoxic drugs containing DNA-damaging agents. The agents inhibit, with some selectivity, proliferation of malignant cells. Another frequently utilized localized treatment modality is radiation therapy (RT). Approximately 50% of all cancer patients receive RT during their course of illness.^5^ In RT, malignant cells are exposed to high-energy radiation, either from externally or internally localized radiation sources. DNA within the exposed cells will be damaged by the high-energy radiation, thereby blocking the ability of these cells to divide and proliferate. With extensive damage to its DNA, the cell eventually dies. Although this is desirable for malignant cells, RT can also significantly impact the surrounding healthy tissues in close proximity to the tumor. Proton- and other hadron-based therapies, rely on the presence of a rather localized Bragg peak for energy deposition and provide a higher damage selectivity, but they are expensive and not widely available.^6^

Consider, for example, the range of treatment strategies for patients presenting with breast cancer. Treatment plans are tailored for patients based on the expression of the estrogen receptors (ER) and progesterone receptors (PR), HER2 protein, the size of the breast tumor, and degree of lymph node involvement.^7^ The first line treatment in the majority of cases remains surgical resection. In particular, for non-metastatic breast cancer, surgery is always recommended to eradicate all tumor cells in addition to sampling and/or removing the axillary lymph nodes with the aim of avoiding metastatic recurrence. Surgical resection is often supplemented by either chemo or hormone therapy. This additional treatment is selected based on whether the ER and PR are positive or negative and may be administered preoperatively (neoadjuvant treatment) and/or postoperatively (adjuvant treatment). Neoadjuvant therapies are provided in an increasing number of locally advanced cases to reduce both the tumor mass and the risk of spread prior to surgery.^8^ Furthermore, RT may also be used to treat a portion of, or the entire, tumor-involved breast (after lumpectomy), the axillary lymph nodes (when their involvement is considered a risk) and the chest wall (after mastectomy). Despite major advances in treatment management over the past decades, refractory diseases and recurrence remain potential problems, while psychological and physical side effects present a significant burden to patients.

In the effort to find treatment options with less frequent and less severe side effects, a majority of the emerging treatment modalities assessed for malignant diseases today are more targeted, localized therapies with minimal damage to healthy cells.^9^^–^^12^ By having a better selectivity to the malignant cells in these schemes, it is also possible to locally increase the treatment dose and thereby more efficiently eradicate a higher fraction of the malignant cells. We find interstitial photodynamic therapy (IPDT) (i.e., intratumor light delivery) to be a promising modality among these localized treatment alternatives under clinical evaluation for some malignant tumor types.

Photodynamic therapy (PDT) is a minimally invasive treatment modality of diseased tissue structures that uses light-activated photosensitizers for localized tissue eradication. Following the pioneering research of Dougherty, Malik, Moan, Pottier, Kennedy, and van den Bergh (i.e., Refs. 13–20) among others, it has been approved for a variety of superficial tumor indications, such as, for instance, non-melanoma skin cancers. Many examples of studies utilizing the particularly promising sensitizer precursor δ-amino levulinic acid (ALA) can be found in the literature.^21^^–^^24^ For larger and/or deeply localized tumors, treatment can be provided via interstitial light illumination using fiber optics. A severe challenge in such a treatment is delivering a sufficient treatment dosage to all malignant cells, while sparing the healthy tissue in the vicinity of the tumor. It is worth mentioning that, even without sophisticated light dosimetry, IPDT is more selective to malignant cells than RT due to the selectivity of the photosensitizer. Moreover, light is strongly attenuated in tissue and is thus confined to a relatively small volume surrounding the fiber optic delivering the treatment light. This yields a localized treatment but also provides a challenge of reaching all malignant cells with a sufficient light dose. A remedy is clearly to use multiple fiber optical probes.^25^ Much of the research to improve IPDT has also been directed toward developing targeted photosensitizers.^26^^–^^29^ Tumor therapy based on IPDT is yet to be fully accepted, awaiting full implementation of refinements, e.g., dosimetry to allow its intrinsically advantageous features to be fully exploited. Returning to the particular case of breast cancer management, IPDT may offer an effective minimally invasive treatment alternative with minimal scarring, thus being an attractive option when considering the increasing concerns of patients with respect to cosmetic results following treatment of low-risk tumor. In addition to breast cancer, a disease with strict treatment options that follows very rigorous protocols, there are several other relevant indications in which IPDT could prove beneficial to the patients, e.g., primary and recurrent prostate cancer.

**Fig. 1:**
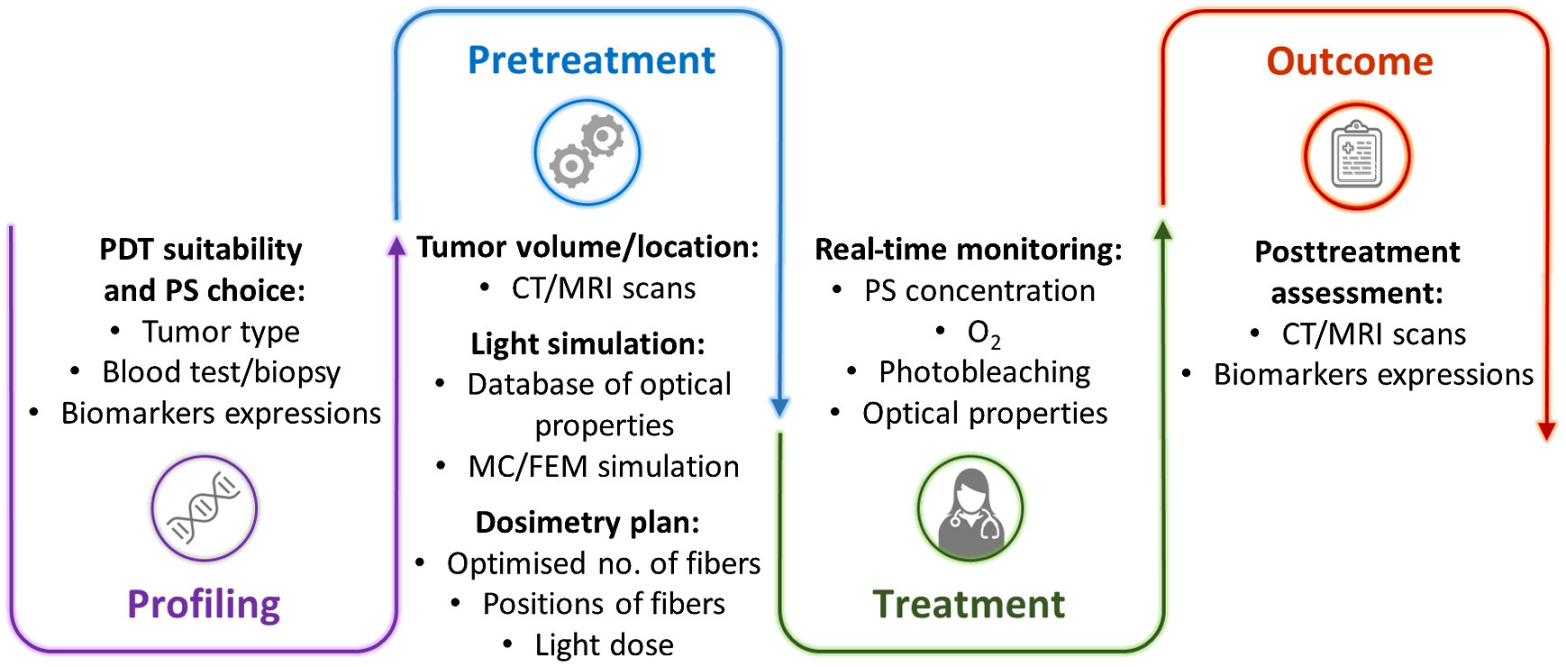
IPDT framework comprising four stages: initial profiling, pretreatment planning, treatment delivery and real-time feedback, and posttreatment outcome assessment.

The purpose of this perspective paper is to explore the potential role of IPDT in the management of malignant tumors and discuss its ability to reduce complications by increasing the selectivity, precision, and safety in tumor eradication. Figure 1 illustrates a perspective of the framework of IPDT that will be discussed. Moreover, adding to previous reviews of the field,^30^^–^^36^ this paper also aims to provide insights into potential future directions for the scientific community as a stimulus for innovation with particular attention focused on the challenges in further improving the IPDT efficacy and tumor selectivity. This could include improved light delivery, dosimetry, and instrument miniaturization for better adaption to the clinical workflow.

## 2 Applications and Challenges for Interstitial Photodynamic Therapy

The potential of IPDT to treat various solid tumors has been investigated in an ongoing process and has demonstrated varying levels of success. In this section, we will describe some of the most promising clinical indications in which advances in IPDT can provide clear benefit in terms of decreased morbidity and increased quality of life.

### 2.1 Central Nervous System Tumors

Through examining the historical development of clinical PDT, one of the earliest clinical applications of PDT was the treatment of glioblastoma multi-forme (GBM) following surgical resection using an intracavity balloon (or similar device) to “sterilize” the tumor margins.^37^^–^^41^ The main goal of this approach was to achieve a reduction in the extent of resection and to either prevent reoccurrence (which is almost inevitable with GBM due to their infiltrative growth) or to extend quality of life/progression-free survival for the patient. These early studies were performed with some form of porphyrin derivative such as benzoporphyrin derivative^42^ or hematoporphyrin derivative (HpD)^37^ or in later studies with talaporfin sodium^39^ or protoporphyrin IX (PpIX) build-up following administration of ALA.^43^ The main initial drawbacks of this approach were patient complications, including increased intracranial pressure (ICP) due to edema, and difficulty in the treatment of very large resection beds or deep tumors, which were not particularly feasible. One possible approach, so far not being implemented, would be to introduce thin plastic tubes with the help of needles through skull burr holes and use these transparent tubes for combined light administration and pressure relief, following the IPDT session. The tubes might also be used for localized administration of sensitizer.^44^

Since the early studies, further work has been carried out from both a PDT perspective and photosensitizers’ perspective that have made PDT at least a compelling adjunct to surgical resection. As described by Stepp and Stummer,^45^ PDT combined with fluorescence-guided resection (FGR, with the EMA and FDA approved ALA-PpIX) led to increases in median survival time and progression free survival. Selectivity between tumor and normal brain tissue, related to blood–brain barrier issues, was very early demonstrated for photofrin and ALA-PpIX, which have very attractive fluorescence properties,^46^ albeit with different properties in that they may not be interchanged with each other. Other research groups have examined IPDT as a possible modality to treat advanced or unresectable GBM with promising results; however, to date, this has not yet led to widespread adoption in many centers.^47^^,^^48^ Thus the promise of PDT for the treatment of GBM still needs to be further substantiated. With the immunological effects of PDT^49^^–^^51^ being explored and preplanning and dosimetry systems being increasingly developed, there is a promising future for this technique. In this section, we will present our perspectives on the future of IPDT with respect to central nervous system (CNS) tumors with a specific focus on GBM along with the challenges associated with the development.

Treatment of GBM with PDT has normally occurred as either postresection sterilization or as an interstitial delivered treatment without surgery. Currently, there are two ongoing clinical trials including GL-01, a pilot phase II trial examining efficacy of stereotactic IPDT (ClinicalTrials.gov #NCT03897491), and NOA-11, a multi-centre, randomized non-blinded trial of stereotactic IPDT for recurrent glioblastoma (ClinicalTrials.gov # NCT04469699). In addition, promising results have recently been published in regards to IPDT for recurrent malignant glioma treated with ALA.^52^ Following surgery and during recovery, combining posttreatment sterilization with implantation of light emitting diodes (LEDs) could help in maintaining an immune-modulator effect through low-dose PDT that induces apoptosis.^53^ Although not strictly IPDT in the classic sense of the term, the goal here is to combine some of the ideas of metronomic PDT^54^^,^^55^ with the advances in dosimetry previously demonstrated by a number of research groups.^30^^,^^48^^,^^56^^,^^57^ Metronomic PDT is compelling in this picture because the low-dose, longer term treatment should lead to an immune-modulator effect while possibly avoiding the complications of raised ICP seen in some instances post-PDT. Furthermore, wireless powered LEDs have been developed and tested (preclinically) with ALA-PpIX for use in metronomic PDT with 8 h long sessions performed daily for 5 days.^58^ A similar idea exists for the treatment of GBM whereby, following FGR, daily administration of PDT could occur at the bedside while the patient recovered postsurgery. These implantable devices could either be removed after the initial treatment period or left in place for possible future applications (similar to deep brain stimulation devices).

Furthermore, these implantable LED devices could contain some form of online or real-time dosimetry that would help guide treatment during these sessions to maximize the overall prescribed dose across the entire treatment volume during the metronomic PDT session. Dosimetry could either target singlet oxygen generation (with interactive steering of the LED operation) or use existing approaches such as photosensitizer bleaching or light fluence monitoring, with the data being entered into a PDT threshold model to calculate the PDT dose for each session. It could be further envisioned that a culture of tumor cells could be grown and treated with different photosensitizers prior to commencement of IPDT to select one with the best selectivity/sensitivity.

The challenges here are both technical and procedural with the development of an implantable non-ferromagnetic/non-metallic LED system that remains roughly in place for a period of 5 days while the tissue recovers postsurgery (with changes in swelling and scarring). Here an expandable or contractile cage may be necessary to contain the LED system. Furthermore, ongoing treatment monitoring with magnetic resonance imaging (MRI) may be necessary to prevent adverse patient outcomes or to monitor LED placement, leading to increased ongoing costs, whereas neuromonitoring would be performed on an ongoing basis at least for the treatment period. Finally, it is possible that many of the photosensitizers utilized clinically may be less efficacious in treating the GBM cells that have invaded normal brain parenchyma, which would necessitate development of new photosensitizers. One particularly exciting development in this field is the use of fibrin glue containing photosensitizer leading to local delivery following surgical resection.^59^ Nevertheless, all of these challenges are tractable and would lead to development of a compelling platform for the treatment of GBM and other brain tumors in the future. Some of the features of IPDT for the treatment of GBMs together with other highlighted applications are summarized in Table 1.

**Table 1:** Specification for IPDT treatment targeted at solid tumors in suitable cancer types.

Parameters	Brain tumors^43^^,^^47^^,^^48^	Prostate tumors^30^^,^^60^^,^^61^	Head and neck tumors^62^^–^^66^
Treatment time	∼up to 60 min (30 min add-on to FGR)	∼up to 30 min	∼up to 10 min
Critical structures nearby	Neural tissue, arteries, veins, and sinuses	Nerves, sphincter muscles, and the rectal wall	Cranial nerves, sensory organs, coronary artery, and brain
Photosensitizers and their dose	Photofrin (2 to 5 mg/kg), ALA (20 to 30 mg/kg)	Temoporfin (0.15 mg/kg), MLU (2 mg/kg), padeliporfin (2 to 6 mg/kg), and verteporfin (up to $15\;{\mathrm{mg}/m}^{2}$)	Photofrin (2 mg/kg) and temoporfin (0.15 mg/kg)
Illumination geometry	Isotropic (with intracavity balloon) cylindrical diffusers	Flat-cut, isotropic diffusers, and cylindrical diffusers	Flat-cut/cylindrical fibers
Side effects	Increased ICP, photosensitivity, and deep vein thrombosis (Photofrin)	Photosensitivity, urinary tract complications, and rectal complications	Photosensitivity, facial edema, coughing, and trouble swallowing
Benefits	Relatively simple integration into surgical workflow	Minimal effects on functional outcomes	Good cosmetic outcome and improved life quality
Challenges	Treatment of brain adjacent to tumor usually inadequate and limited penetration depth	Personalized dosimetry	Personalized dosimetry and lack of comparative study to SoC

### 2.2 Prostate Cancer

Due to the high incidence of prostate cancer, light-induced therapies such as PDT have been increasingly studied in the last four decades as alternative and co-adjuvant strategies for improving patients’ outcomes. Primarily four photosensitizers have been used in clinical work in PDT for prostate cancer: temoporfin,^30^^,^^67^ motexafin lutetium (MLU),^68^ verteporfin,^69^ and padeliporfin.^61^ Of these, the two latter are in active clinical programs as of 2021 with padeliporfin (Tookad®) being the only photosensitizer currently approved in some countries for prostate cancer PDT. In prostate PDT, the general approach used in most work is similar to brachytherapy of prostate cancer. This means that transrectal ultrasound (TRUS) imaging is used as the basis for dose planning, and a brachytherapy template is used to guide optical fibers to the target volume, transperineally via a set of cannulas, to predetermined coordinate points in the tissue. The different groups active in this research field have adopted slightly different strategies for dosimetry. PDT dosimetry for prostate cancer is especially important because of the proximity of nerves, sphincter muscles, and the rectal wall, all of which can give rise to complications if over-treated. At the same time, the optical properties of prostate tissue are likely to be fairly varied when considering the entire range of the patient population and the different disease scenarios: age, size of prostate, disease grade, amount of calcification, and whether the tissue has been radiated or not. This implies that personalized dosimetry is required for full optimization of treatment parameters. On the other hand, the capsule surrounding the prostate is made up of fibromuscular tissue that may provide some optical shielding effect to the surrounding structures.

Dose planning can either be done empirically, with a preset light dose per optical fiber or per cm diffuser,^60^ or based on real-time measurements of the optical properties and model-based calculation of the optimal dose per fiber.^70^ With the former approach, successful results have been demonstrated in the context of Tookad^®^ treatment, in which diffusing fibers were inserted at positions recommended by radiologists and urologists aided by treatment guidance software.^61^ A fixed light dose of 200  J/cm was then delivered per fiber. Although both approaches have resulted in successful outcome in clinical trials, there are optimizations in terms of dose planning and dosimetry that could be carried out in future work, which could lead to even better tumor management and/or a reduced risk of side effects. In clinical trials, the most advanced dosimetry paradigm to date has been based on the assumption of a light dose threshold to achieve ablation. There are PDT laser devices capable of monitoring tissue oxygen saturation and photosensitizer fluorescence,^30^ but so far these parameters have not been used for real-time feedback of the light dose in the treatment situation. There is a clear opening in future clinical work to develop a PDT dosimetry model that closes the feedback loop with local tissue oxygenation and photosensitizer fluorescence, the latter being related to the concentration of photosensitizer. This expected near-time development would allow for fully harvesting the fruits of interactive IPDT in individualized patient treatments. Posttreatment reconstruction of 3D mapping of the photosensitizer signal in prostate has been demonstrated.^71^

### 2.3 Head and Neck Malignancies

The potential benefits of PDT for the treatment of head and neck cancers (HNC) have been evaluated since the 1980s.^72^ Despite its demonstrated effectiveness,^73^ PDT has not yet been widely adopted in clinical practice as a conventional line of treatment in the case of HNC. HNC represent a heterogeneous group of tumors with large variations in etiologies, anatomical locations, prognoses, and tumor stages and are known for their high morbidity and aggressive behavior.^74^ Effective management of such a diverse and complex group of cancers demands a multi-modal approach combining surgery, radiation, and chemotherapy. In addition to treatment efficacy, the proximity of several critical structures such as cranial nerves, sensory organs, major vessels, and the brain may cause treatment related physiological dysfunctions, and the location of the tumor could lead to facial disfigurement. Therefore, it is expected that any future treatment advances would improve both the patient’s survival and the subsequent quality of life.

The most common malignancies that arise in the head and neck area, accounting for almost 90% of cases, are head and neck squamous cell carcinomas (HNSCC), the annual incidence of which continues to increase worldwide.^2^ Conventional PDT is ideally suited for treating superficial (<5  mm deep) early stage lesions. A number of studies, mostly in oral cavity and larynx, have indicated that it can be successfully used to treat stage I/II SCCs as a primary modality using porfimer sodium (photofrin), hexyloxyethyl-devinyl pyropheophorbide (HPPH), or meta-tetrahydroxyphenylchlorin (temoporfin, Foscan) as photosensitizers, with results comparable to surgery, while allowing for preservation of functions such as voice or swallowing.^75^^–^^78^ Building on the positive patient responses, relative simplicity, and low cost of this approach, PDT is also showing great promise for treating early stage oral cancers as a primary modality in developing countries with high incidence.^79^^–^^81^

On the other hand, 60% of the patients present with already advanced HNSCCs (stage III/IV). Recurrent and metastatic cases often exhibit the additional challenge of acquired cytostatic drug resistance. For patients who have exhausted conventional treatment options, IPDT provides an alternative modality that may allow for an improved quality of life and prolonged survival. Interstitial treatment has been reported with both photofrin and temoporfin in a number of retrospective studies^82^ and a large multi-institutional prospective study (temoporfin only),^62^ but little randomized data are available. A new generation photosensitizer, redaporfin, with increased absorption and improved reactive oxygen species (ROS) generation, is also being evaluated in treatment of advanced HNC.^83^ Careful IPDT treatment planning is required to ensure that a therapeutic dose is delivered to the tumor while minimizing damage to surrounding healthy tissue. In addition to standard imaging approaches such as computed tomography (CT) or MRI, which are typically used for pretreatment planning,^84^ intraoperative ultrasound (US) is being investigated as a guiding tool for the insertion of the fibers during IPDT.^85^ Modified brachytherapy techniques have been used for HNC treatment planning^65^ and were found to be useful in guiding physicians in decisions on the number of fibers, although without information on light fluence distribution. Another approach used graphical processing enhanced Monte Carlo (MC)^86^ simulations or finite-element methods^87^ to model light delivery in near real time. Improved dosimetry techniques with standardized protocols would benefit the outcomes of the treatment and help to gain wider acceptance within the clinical community.

Interestingly, recent research studies in HNSCC cell lines have assessed the role of human papilloma virus (HPV) in the responsiveness of tumors to radiation and PDT therapy.^88^^,^^89^ Clinical characteristics of HPV-associated HNSCC include its greater sensitivity to radiotherapy and better survival of patients compared with those with HPV-negative HNSCCs. It has been found that PDT directed at the endoplasmic reticulum/mitochondria induces a cell-death mode called paraptosis. This leads to a significant increase in radiation response in intrinsically radio-resistive HPV-negative tumors. Photofrin, temoporfin, and HPPH, photosensitizers typically used in HNSCC treatment, target sites including endoplasmic reticulum and therefore can initiate paraptosis. Although further studies are needed, this shows great promise for PDT serving as a radiosensitizer in HPV-negative HNSCCs.

The main advantage of PDT treatment of HCN—increase in the quality of life—has been demonstrated in the past; however, studies of efficacy compared with conventional treatment options are currently still lacking. New prospective trials should aim to illustrate better the added value of this treatment modality by conducting systematic assessments of life quality before, during, and after PDT therapy. Furthermore, the combination of FGR with IPDT for advanced HNSCCs would enable clinicians to push the envelope into more precise surgical approaches leading to improved outcomes and subsequent improved life quality. In a quest for targeted delivery methods for improved selectivity, various nanoparticles (NPs) as a carrier system for photosensitizers are also being explored.^90^^,^^91^ This could have the potential to expand the application of PDT for large HNSCCs.

### 2.4 Breast Cancer

As mentioned already in Sec. 1, another interesting application of IPDT is mammary cancer, which is the second most common appearing female cancer in the Western world after skin malignancies. The incidence in many countries reaches as high as 1 out of 10 females with an increasing tendency due to social and lifestyle factors.^92^ There is a clear relation to the use of hormone therapy (anticonceptual drugs in early age), late menopause, first pregnancy at late age (more than 35 years), shorter period of breast feeding, and other factors related to urban lifestyle.^93^ As noted, the standard procedure with or without neoadjuvant chemotherapy is surgery. The tendency in surgical treatment has gone from radical therapy (mastectomy) to more tissue sparing procedures with sector resection. Even so, surgery leaves scarring in the tissue and in some cases a re-resection has to be performed due to residual tumor margins. This means that surgery disturbs the natural process of healing. Beside the physical adverse effect, it also causes the patient enormous physiological anxiety with a second round of treatment close in time to the primary procedure. With optimal dosimetry, IPDT could be an attractive alternative with the non-invasiveness advantages disturbing the tissue in a minimal way. Promising results have been reported with verteporfin in an initial study of primary breast cancer in a limited number of patients (15 patients).^94^ According to the results, evaluated either with histology or MRI, necrosis was achieved in the treatment volume, while apoptosis was evident in the adjacent tissue due to the inflammatory response of PDT. The study was performed with one diffusing optical fiber inserted under the US guidance. With a multi-fiber arrangement and refined light dosimetry, the circumstances could certainly be further improved.

Another challenge in breast cancer is the surgical dissection of the axillary lymph nodes. The conventional surgical procedure has a certain degree of side effects with a swollen and tight-feeling arm, sometimes due to a fluid-filled seroma or obstructed lymph node drainage; stiffness of the arm and shoulder; and changes in sensation with or without pain.^95^ Also for this indication, a multi-fiber IPDT approach with optimal dosimetry could certainly be a valuable niche following clinical evaluation.

### 2.5 Other Applications

In addition to the CNS, prostate, HNC, and breast tumor fields, IPDT has been investigated for a number of different clinical indications with some success. Although it is out of the scope of this paper to review all of these indications, we will focus on a couple of applications in which IPDT can have a positive impact in the management of tumors. The first of these instances is for intrathoracic tumors and specifically malignant pleural mesothelioma. Although a relatively rare tumor, its incidence has been increasing recently due to previous asbestos exposure,^96^ and it carries a particularly poor prognosis with a median survival time of 8 to 14 months.^97^ PDT has been used previously with success in small clinical trials.^98^^–^^100^ Furthermore, many dosimetry methods have already been investigated and integrated into the treatment platform. These dosimetry methods have included measuring photofrin photobleaching,^100^ ROS production,^98^ and light fluence, to name a few.^99^^,^^101^

For this indication, IPDT could play a further role in treatment and possibly expand the number of treatment sessions or lengthening the treatment out beyond just using IPDT as an adjunct treatment postsurgery. With 3D printing technologies useful for placement of dosimetry sensors into the intrathoracic cavity based on preoperative/perioperative imaging and more advanced MC simulation algorithms, fibers could be placed interstitially postsurgery under US, endoscopic, or infrared navigation guidance^102^ through a thoracotomy port for treatment. Dosimetry could be modeled based on postoperative CT or MRI and then a number of fibers and light fluence per fiber could be calculated *a priori*, similar to the planning phase described in the dosimetry section below. Further development of novel photosensitizers, such as folate-targeted porphyrin lipids,^103^ should further increase the therapeutic efficacy of the treatment.

A second indication for the use of IPDT that has gained adoption again recently is the treatment of non-muscular invasive bladder cancer.^104^^,^^105^ Utilization of IPDT for the treatment of bladder cancer was investigated over a number of years.^106^^–^^108^ Recently, research groups have explored the use of integrated dosimetry cages with PDT fibers that expand within the bladder cavity for whole bladder irradiation. This approach was first examined in a phase I clinical trial that utilized integrated light fluence sensors that continuously monitored both the fluence and total dose. Once the threshold dose was achieved in a predetermined number of sensors, the treatment was terminated.^56^

This approach could be further developed and lead to a more personalized treatment for patients, based on preoperative diagnostic imaging. Optimal placement of both light sources and detectors for optimal coverage of the tumor and more diffuse placement for whole bladder coverage can be sought. Such an approach, within the bladder, would require specific orientation of the cage and this could be achieved utilizing fluorescent navigation (including using ALA-PpIX^105^ or another fluorescent photosensitizer^109^) or through direct measurement of optical properties. This could provide benefits on several levels including reducing the complications of damage to normal bladder wall and the underlying muscular *propria*, reducing over-treatment of normal bladder epithelia while possibly under-treating the tumor itself, and further enhancing the dosimetry of the treatment. Furthermore, a personalized approach with real-time monitoring could also allow for a variety of different photosensitizers to be used based on preoperative MC simulations, tumor depth of invasion (superficial versus invasive), any nearby critical structures, relative volume of tumor, and blood vessels within tumor. One potential issue that would need to be solved is the reduction of the turbidity of instilled water during the procedure, which leads to false light fluence values during treatment. This may be solved by a continuous infusion of water through the working channel of the cystoscope, albeit this is not ideal from a surgical or cost perspective. However, this does not present as an absolute impediment to an elegant use of IPDT in this application moving forward.

## 3 Integration of Dosimetry in IPDT

As illustrated in Fig. 1, dosimetry in IPDT is of key importance due to the need to ensure that the tumor is completely treated, while at the same time avoiding over-treating surrounding organs at risk. The large variability in the outcome of some clinical studies on IPDT may be due to the failure to properly address dosimetry aspects. Since the clinically approved photosensitizers have limited or no cancer tumor specificity, light dose control and dosimetry are important parts of clinical IPDT design. Practical dosimetry approaches always start with medical images as input to dose planning and optical fiber placement. The methods to navigate the optical fibers to the desired positions in the tissue are closely related to those used in interventional radiology.

Because of the variation in tissue properties between individuals, in different parts of the treated tissue, and over time during the course of the treatment, monitoring of tissue parameters is crucial. At the scale relevant to PDT dose (millimeters), light attenuation is determined by the local absorption coefficient $\mu_{a}$ ($m^{-1}$), scattering coefficient $\mu_{s}$ ($m^{-1}$), and dimensionless scattering anisotropy factor g. In the diffuse light propagation regime, it is common to use the reduced scattering coefficient $\mu_{s^{\prime }}=\mu_{s}(1-g)$. Often, these coefficients are combined into a single parameter, the effective attenuation coefficient $\mu_{\mathrm{eff}}={\left[3\mu_{a}(\mu_{a}+\mu_{s^{\prime }})\right]}^{1/2}$. Estimation of these optical properties is based on optical measurements, performed interstitially or at the boundary of the tissue region of interest. This may be done using the same optical fibers as used for the therapeutic light delivery^30^^,^^110^ or using a different set of light sources and detectors.^111^^,^^112^ The optical properties are estimated by means of an inverse method in which the measurement data are fitted to a light propagation model.

The optical fibers used to deliver and measure the light may be bare-end or have some kind of diffuser at the tip. Bare-end fibers have the advantage, in comparison with extended diffusers, of the evaluation of optical properties being easier since light sources and detectors can be treated as points. It also potentially provides higher spatial resolution. On the other hand, cylindrical diffusers allow for using fewer optical fibers to cover the same treatment volume, and it has been recently shown that cylindrical diffusers can be used to evaluate the optical properties.^113^^,^^114^

The measurements can be done in continuous wave (CW) mode or using pulsed (∼ps short pulses) or frequency-modulated (∼100  MHz) light. Generally, modulated measurements may provide more information, which can give more accurate predictions, but the technology is more complicated and costly. In particular, using modulated light, it is possible to separate the effect of local inhomogeneities close to the optical fibers inside the tissue from the optical properties of the bulk medium.^115^ With CW data only, it may be difficult to achieve a similar distinction. Also by modulation frequency tagging, all measurements can be done simultaneously instead of sequentially.

Other tissue parameters of importance for PDT dosimetry are the photosensitizer concentration, tissue oxygen saturation, and singlet oxygen production. The photosensitizer concentration can be estimated by optical methods based on fluorescence emission.^71^^,^^110^^,^^116^^–^^121^ Fluorescence excitation by short wavelengths (∼400 to 600 nm) may be difficult to achieve in the interstitial setting because of the very limited light penetration length. Fluorescence excitation at wavelengths above 600 nm, on the other hand, can be utilized for photosensitizer agents that exhibit absorption in this wavelength region. Some model-based methods directly determine the concentration of the photosensitizer in the tissue based on fluorescence, which is hard to achieve since it requires absolute-calibrated detection and knowledge of fluorescence quantum yield as well as quenching effects in the tissue, in addition to knowledge of the optical properties. Tomographic reconstruction of the relative fluorescence signal throughout a tissue volume has been performed^71^ and provides a scheme for estimating spatial distribution of the photosensitizer within the prostate. In addition, tissue oxygen saturation can be determined by spectral fitting methods in the near-infrared region, where the differences in the absorption spectra of deoxyhemoglobin and oxyhemoglobin are significant.^110^^,^^119^^,^^122^^,^^123^

**Fig. 2:**
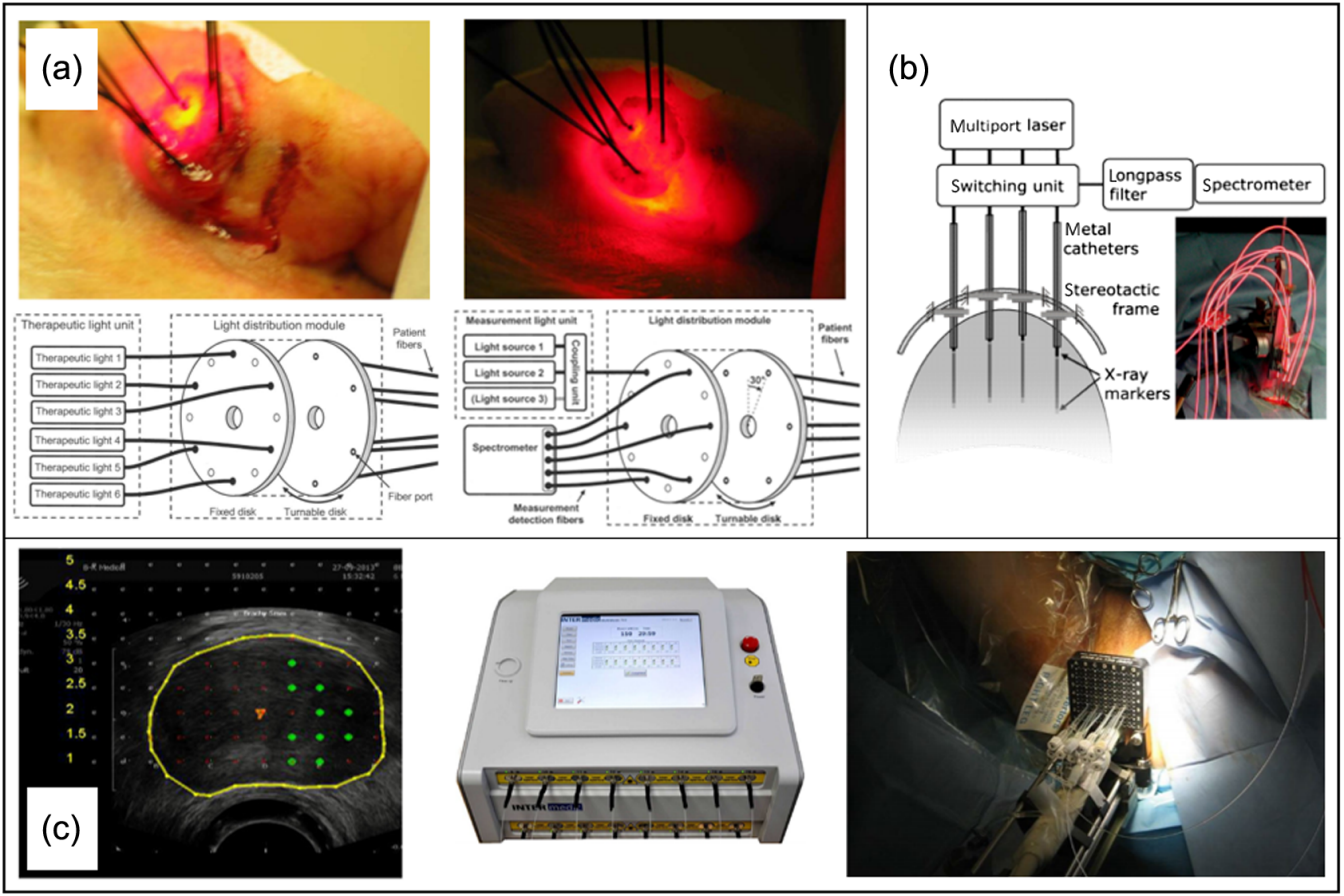
(a) Six-fiber IPDT system with diagnostic and treatment capability: top panels present diagnostic mode (left) and treatment mode (right) of operation; bottom panels depict the diagrams of fiber arrangement for the diagnostic (left) and therapeutic mode (right). Reproduced with permission from Ref. 33, courtesy of SPIE and OSA. (b) IPDT setup with real-time spectroscopic monitoring for brain tumors. Inset shows a photograph of the clinical setting during IPDT procedure. Reproduced with permission from Ref. 48, courtesy of Wiley. (c) IPDT prostate treatment: guidance with TOOGUIDE software (left), dedicated laser generator (middle), and preoperative view of vascular-targeted photodynamic therapy in action (right). Reproduced with permission from Ref. 131, courtesy of Springer Nature (based on CC BY license).

Several approaches to conduct dosimetry plans to guide IPDT have been proposed.^48^^,^^70^^,^^110^^,^^112^^,^^124^ More generally, dosimetry models can be categorized into explicit dosimetry, implicit dosimetry, direct dosimetry, and biological response, with their respective advantages and constraints described broadly in the literature.^31^^,^^125^^–^^128^ Table 2 summarizes some of the recent dosimetry systems proposed for clinical use by various groups, whereas Fig. 2 presents examples of systems with pretreatment planning and online dosimetry. All dosimetry methods presented in that table are based on a light dose threshold model, specifically determined in these cases, and depend on the photosensitizer, drug dose and its formulation, drug–light interval, and clinical indication. Here we will use one set of algorithms to illustrate the considerations needed to develop IPDT treatment planning and dose control, referred to as “Interactive Dosimetry by Sequential Evaluation” (IDOSE).^70^ In the first step, specific to prostate cancer PDT, US images are acquired by TRUS. Based on the images, a computer model is generated with the key tissue types: prostate, urethra, rectum, and sphincters. In the next step, the positions of the optical fibers are calculated using a random-search optimization algorithm, similar to simulated annealing-type algorithms. The algorithm uses a light propagation model based on the diffusion equation and assumes that the prostate tissue is homogeneous with average optical properties.

**Table 2:** Comparison of some approaches and systems proposed for IPDT dosimetry.

Method	Fiber type and number	Tumor type	PS/dose/drug–light interval	Treatment dosimetry and control
PDT-SPACE^124^	Cylindrical diffuser	Synthetic brain tumors	Various modeled	• MRI-based treatment planning method
				• Provides optimized power allocation based on light distribution computed by FullMonte and power allocation algorithm for cylindrical light diffusers
				• In comparison with Cimmino’s algorithm, it improves the preservation of organs at risk
Johansson et al.^48^	Four to six cylindrical diffusers inserted using stereotactic approach	GBM	ALA, 30 mg/kg 5 to 8 h	• Real time fluorescence monitoring for treatment prognosis
				• Measurements of PpIX fluorescence intensity and photobleaching efficiency prior to and during treatment
				• Constant power of 200 mW per fiber until dose 720 J/cm per fiber reached
IDOSE^70^	Up to 18 bare-end fibers inserted under US guidance	Prostate cancer	Temoporfin 0.15 mg/kg 96 h	• Real time dosimetry based on light dose threshold model
				• Provides light dose plan (calculated from diffusion equation and optimized by Cimmino method) with optical fiber positions based on 3D tissue models from US from diffusion equation
				• At specific intervals monitoring of light fluence is performed and dose plan is updated
				• Constant power of 150 mW per fiber, light delivery time varied for each fiber depending on measured light dose delivered
Davidson et al.^129^	Four to six cylindrical diffusers inserted with US guidance	Prostate cancer	Tookad 2 mg/kg 6 min	• MRI-based prospective PDT planning
				• The dosimetry concentrated on the light optical properties (diffusion model fitted to *in vivo* fluence measurements) and the light fluence delivered to various regions of the prostate
				• 62% of patients with light dose $>23\;J\;{\mathrm{cm}}^{-2}$ had complete biopsy response
				• Constant power of 200 mW/cm for 30 min
TOOGUIDE TRUS^60^^,^^130^	Up to 21 cylindrical diffusers inserted with US guidance	Prostate cancer	Tookad 2 to 6 mg/kg	• MRI-based platform for pretreatment dosimetric planning
				• Uses Powell’s algorithm to provide optimization of number of fibers and fiber position and length
				• Constant power of 150 mW/cm for 1333 s
				• The model is based on the correlation between the necrosis volume and the volume illuminated by the light diffusers used
				• Correction of the swelling factor

When the fibers have been put in place, but before PDT light delivery starts, a series of measurements is performed to collect the value of the light attenuation between each mutual pair of fibers. These measurements are fed into an inverse algorithm that fits the data to a light propagation model based on the diffusion equation, with the aim of determining the local attenuation coefficient $\mu_{\mathrm{eff}}$ around each fiber. The approach is feasible since the same set of fibers can be used for diagnostics as well as treatment. As a matter of fact, this is also the case regarding oxygenation and sensitizer distribution assessment.^25^^,^^30^

A second optimization algorithm is used to calculate the light dose to be delivered from each fiber. The algorithm is based on Cimmino’s method. Briefly, the dose given to each tissue voxel in the tissue model is calculated using a diffusion model, by summing the contribution from all fibers. The values of $\mu_{\mathrm{eff}}$ (for each voxel) from the previous step are used as input to the diffusion model. The emitted light dose from each fiber represents the optimization parameters. Each tissue type is assigned a dose threshold value, with the aim of reaching at least the threshold dose in the target region, while avoiding reaching the threshold dose in all other tissue types. Each tissue type can also be assigned a weight coefficient depending on its importance. This leads to a set of inequalities that can be solved by Cimmino’s method. The implementation uses the block-action method described by Censor et al.^132^ The method is iterative and converges toward a solution even if all constraints are not met.

**Fig. 3:**
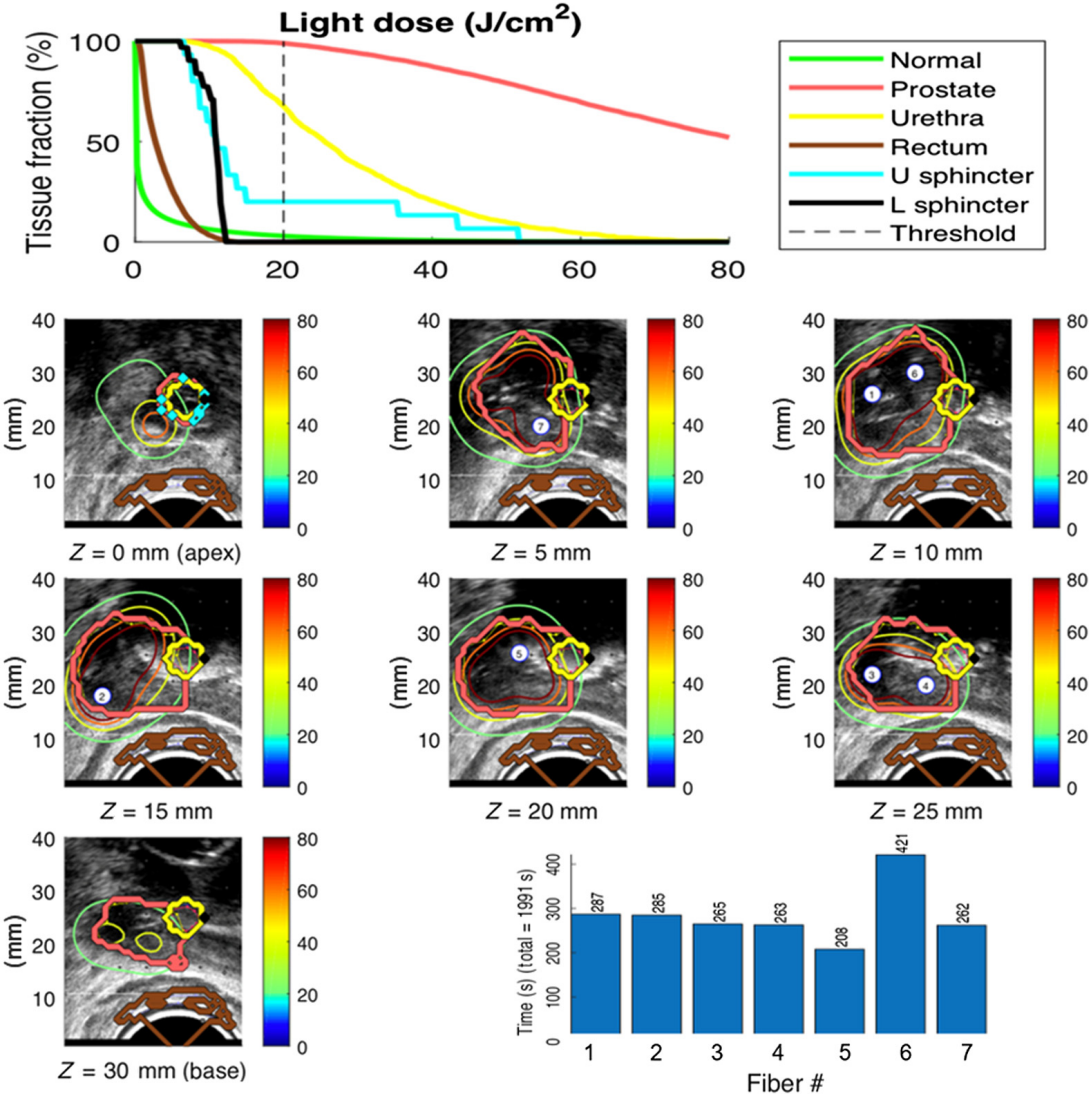
Illustration of dose plan for focal prostate PDT, in which the right posterior side of the prostate is targeted using seven bare fibers as sources. The dose unit is $J/{\mathrm{cm}}^{2}$. The upper chart shows the DVH with the dose threshold indicated at $20\;J/{\mathrm{cm}}^{2}$. The images show the isodose curves overlaid on US images and segmentations of prostate (in red), urethra (in yellow), and rectum (in brown). The lower right chart shows the total light illumination time durations per fiber.

During the PDT session, light delivery is interrupted at intervals to acquire new measurements, which are again evaluated by the inverse fitting algorithm. This results in updated values of $\mu_{\mathrm{eff}}$ in case changes have occurred in the tissue due to, for example, variations in blood flow. The $\mu_{\mathrm{eff}}$ values are used to calculate new light doses for each fiber, and an updated dose plan is presented to the user for approval. This cycle is repeated until all fibers have delivered their full dose. The Cimmino algorithm does not allow for straightforward implementation of constraints based on dose-volume histograms (DVHs), but DVHs are used to evaluate the performance of the dose planning. An example of a dose plan from a clinical case is shown in Fig. 3.

IPDT dosimetry is an essential tool in optimal treatment management, ensuring that the appropriate light dose is delivered without over- or under-treatment. Although biological processes governing the treatment response are complex and may seem challenging to control in real time, several approaches show great promise for predictable and reliable dosimetry protocols. In addition, for certain indications (e.g., palliative care in HNC), extremely precise dosimetry may not be necessary to fulfilling the clinical objective.

## 4 Photosensitizers

Photosensitizers are key elements in the PDT approach to tumor management. The ideal photosensitizers must be chemically stable and systemically non-toxic, accumulate in high concentrations in the tumor tissue as compared with surrounding tissue, have high absorption at wavelengths sufficient for deep tissue penetration, have high ROS generation yield, and be cleared from the system rapidly after treatment.^29^ Despite a huge research effort, to date only a relatively small number of photosensitizers have received approval. Photofrin, the first FDA-approved photosensitizers based on an HpD, paved the way for PDT as an alternative cancer treatment in clinics. This first-generation photosensitizer remains widely used in many countries in the treatment of various cancers, despite its low selectivity, limited absorption, and long-term photosensitivity.^133^ However, this has led to the development of second-generation photosensitizers such as chlorins, bacteriochlorins, phthalocyanines, and other porphyrin derivatives in an effort to mitigate these drawbacks. Several second-generation photosensitizers have been approved in certain countries for the treatment of different cancer types such as ALA for skin and brain (EU and North America); temoporfin for HNC, bile duct, and lung (EU); padeliporfin potassium (Tookad) for prostate (EU and Mexico); and talaporfin sodium (Laserphyrin) for lung and brain (Japan), to name a few. The treatment outcomes obtained with these photosensitizers have demonstrated reduced side effects, improved selectivity to the target tissue, and deeper penetration depths thanks to longer excitation wavelengths.^134^

Despite the improvements enabled by the second generation of photosensitizers, several challenges remain to be solved by the next generation of sensitizing agents. First and foremost, new strategies to further increase the selectivity to the tumor cells to improve efficacy and reduce systemic cytotoxicity should be pursued. Recently, emerging third-generation photosensitizers refer to modified second-generation photosensitizers that aim at targeting strategies such as antibody-conjugated photosensitizer binding to receptors over-expressed in tumor cells (active targeting) and photosensitizer-loaded nanocarriers (passive targeting), assisting in the delivery of photosensitizers to the tumor and increasing selectivity versus normal tissue. This should result in a lower photosensitizer dosage, increased allowed light intensity, and fewer unwanted side effects. Several clinically approved monoclonal antibodies have become an appealing option for targeted delivery of anticancer drugs owing to their high target specificity and affinity. The first antibody–photosensitizer conjugate (water-soluble silicon–phthalocyanine derivative IRDye700DX conjugated to Cetuximab) targeting epidermal growth factor receptors has received early conditional marketing approval in Japan for the treatment of advanced HNC^135^ while conducting a global phase 3 multi-center clinical trial (NCT03769506). This delivery strategy is highly versatile as different antibodies can be used to target IR700 to other antigens, which has been demonstrated in preclinical models for breast, brain, prostate, and oral cancers. On the other hand, the fact that most effective photosensitizers tend to be insoluble means that encapsulation in nanodrug carriers could improve their performance. Different organic (e.g., lipids and peptides) and inorganic (e.g., metallic, silica, and quantum dot) NPs have proven successful in *in vitro* and *in vivo* models.^136^ However, the mechanism by which NPs enter solid tumors is more complex than previously thought and the enhanced permeability and retention effect is significantly more pronounced in the small animal xenograft models compared with tumors growing in humans.^137^

The second significant challenge, particularly in the case of solid tumors, is elevated intratumoral pressure and tumor hypoxia (oxygen pressure of <10  mm Hg). Novel ways of overcoming heterogeneous drug uptake resulting from increased interstitial fluid pressure may be important for increasing the efficacy of PDT in certain cases.^138^^–^^140^ The therapeutic efficacy of conventional PDT relies predominantly on the type II mechanism, which requires the presence of three inseparable elements: photosensitizer, light, and oxygen. However, in solid tumors, the already low oxygen concentration is further diminished by the PDT process, leading to low efficacy of the treatment. Therefore, for these applications, oxygen-independent PDT would be beneficial. It has been shown that intermittent light delivery (fractional PDT) allowing for replenishment of cellular oxygen and oxygen delivery strategies can improve treatment outcomes.^141^^,^^142^ Recently, another strategy utilizing type I PDT, which activates free radicals, appeared to be a direct way of overcoming hypoxia limitations.^143^^,^^144^

Development of novel photosensitizers is an active area of research, and the real clinical influence of newer NP-based systems is anticipated in the next decade. With many existing photosensitizers being evaluated in clinical trials and novel strategies to mitigate their potential drawbacks for applications in solid tumors, IPDT has the potential to gain wider clinical acceptance for the treatment of deep-seated tumors in the near future.

## 5 Future Opportunities

The general perspective and potential of IPDT in the management of solid tumors will now be summarized through a subjective SWOT analysis in comparison with other local treatment options for well-selected clinical indications. The main strength of IPDT stems from its minimally invasive nature and the fact that it can prove to potentially be an effective treatment with fewer side effects. The treatment neither targets the nucleus nor the collagen network in the tissue, meaning that it is non-mutagenic and can therefore be repeated as many times as necessary without impairing the integrity of the tissue structure. The tissue also typically heals well after treatment as typically the collagen and lipid structures are not affected and hence scars are only minute. This offers an attractive solution for indications requiring a good cosmetic effect such as breast cancer or HNC. The relatively shallow light penetration can be seen as both a strength and a limitation. Light, and thereby the treatment response, will be confined to a rather small region in the vicinity of the treatment fiber tip, facilitating a selective treatment opportunity. This forced confinement proves useful when it comes to tumors in close vicinity of critical structures such as prostate or HNC. At the same time, the short light penetration is a challenge when treating large volumes, calling for the use of using multiple optical treatment fibers in such cases.

Weaknesses of the IPDT modality include both the elaborate treatment mechanism complicating its planning and optimization and potential side effects of the treatment, which while milder than those of other therapy modalities are still present. The dependence of the treatment outcome on many parameters can be viewed as both a complication and an opportunity as it suggests the need for sophisticated treatment optimization and planning. This implies also the requirement for specifically developed instrumentation^25^^,^^30^ and expertise.^70^^,^^124^ The major remaining side effects of IPDT involve possible pain during and after treatment, treatment response deviating from the plan due to undetected bleeding in connection with placing the optical fibers, and adverse reactions to the photosensitizer given, the latter clearly being a factor pertaining to all types of medication. The mitigation of these side effects is appropriate treatment protocols with suitable dose and type of photosensitizer and the use of multiple fibers to minimize the influence of any bleeding around the fiber tip. Repositioning of a fiber once bleeding is detected (and it can be readily detected in an integrated diagnostic/treatment IPDT system) can be employed to mitigate a reduced dose.

The opportunities, however, for IPDT are very high in handling unmet clinical needs. The complexity of the treatment mechanisms yields many opportunities to optimize and personalize the treatment via advanced dosimetry and targeting photosensitizers. This, together with the few and mild side effects, yields an attractive treatment option for a number of clinical applications as described above. Furthermore, the treatment also provides additional opportunities to utilize biomarkers indicative of PDT outcome when deciding the treatment strategy or monitoring and optimizing the outcome, which still remains a largely unexplored path. This can then aid in selecting IPDT when the modality is well suited and allow for individualisation of the treatment plan depending on prior information obtained from blood samples or tumor biopsies. For example, it has been suggested that ABCG2, FECH, and HO‐1 could serve as indicators of treatment outcome for ALA-PDT of brain tumors, while in HNC, STAT3 could be used as a molecular marker of cumulative photoreaction therapy monitoring.^77^^,^^145^ In addition, the use of multiple fibers, when arranged for an integrated combination of treatment, and monitoring of important data such as light flux, oxygenation, and sensitizer distributions provides excellent opportunities for optimized treatments, as discussed.^25^^,^^30^^,^^70^ Furthermore, a special feature of PDT and IPDT is their adaptability for a realistic and low-cost tumor treatment modality in countries with very low resources and lack of operating rooms and facilities for ionizing radiation.^80^^,^^146^

What then may be a threat or impair a favorable utilization of IPDT for the management of solid malignant tumors? Some of the current challenges faced by IPDT are outlined in Table 3. It could possibly be that the approach would not achieve the expected convincing results regarding treatment outcomes in certain conducted studies. The reason for this may be that full control of important treatment parameters could not be achieved, in view of the fact that the modality, at least in certain cases, would require full control of light dose, sensitizer, and oxygen availability. Suboptimized treatment delivery would obviously result in suboptimal treatment outcomes. Clinical trials with equipment allowing for full control of relevant parameters without causing extra trouble, neither for the patient nor for the doctor, would then lead the way to improved results. It is important to correctly plan studies and translate the results from one study to another to help optimize a study for a different indication, based on prior experience. Clearly, the development of other novel treatment modalities with good outcomes might also challenge IPDT. Such techniques might possibly include immunomodulated therapy. One could then consider IPDT to be part of a combined treatment protocol for tumor debulking.

**Table 3:** Summary of the present challenges and needs of IPDT to be included in clinical guidelines for the treatment of certain indications.

Improvement potential	Present challenges	Need
Photosensitizer	Small number of approved photosensitizers and lack of approved targeting photosensitizers	Highly efficient photosensitizers with very specific uptake in malignant tissue and without side effects
	Dependence on oxygen in hypoxic tumor environments	
Dosimetry model	Spatial mapping of all parameters influencing treatment outcome during the cause of the treatment	Advancement of the model to incorporate all parameters contributing to the treatment outcome
	Dosimetry model including all parameters for inline treatment guidance	
Light fluence modeling	Application of accurate, while slow, light fluence calculations (primarily MC) for use in online treatment control	Accurate model of light propagation that could be used both for pretreatment planning and for online treatment guidance
Light delivery	Determination of how many fibers to employ and how the spatial emission profile looks	To distribute light for optimal DVHs, while also enabling sufficient mapping of the dosimetry parameters measured optically
	Active control of emission profile/location along the distal fiber tip	
	Overcome (or control) deformations due to fiber insertion with inline mapping	
Expand the application area of IPDT	Small number of prospective randomized trials	Collect more clinical data to support definition of clinical protocols for new indications
	Large variability of the results of clinical trials	
	Very few approved indications for clinical use of PDT	

## 6 Conclusions

The potential clinical benefits of IPDT have been clearly demonstrated over the years. However, the modality is still at an early clinical stage with clear opportunities for further development. Treatment selectivity and the low number of adverse side effects present IPDT in a favorable light in comparison with standard modalities used to treat solid tumors. In our perspective, this treatment once fully exploited in clinical settings will extend its reach beyond indications investigated in current clinical trials. Following present approvals of first clinical indications and further considerations regarding optimized dosimetry and suitable photosensitizers fulfilling needs for each individual oncological case, IPDT will gain entry into the treatment guidelines and wider acceptance among the clinical community. Advances in photosensitizing agents, light delivery systems, and treatment planning schemes offer numerous opportunities for this technique to become a robust, standard first-line therapy, either alone or in combination with other treatments, for a variety of malignant diseases.
